# Determination of a prediction model for therapeutic response and prognosis based on chemokine signaling-related genes in stage I–III lung squamous cell carcinoma

**DOI:** 10.3389/fgene.2022.921837

**Published:** 2022-08-31

**Authors:** Jinzhi Lai, Shiyu Yang, Shuqiang Chu, Tianwen Xu, Jingshan Huang

**Affiliations:** ^1^ Department of Oncology, The Second Affiliated Hospital of Fujian Medical University, Quanzhou, Fujian, China; ^2^ Department of General Surgery, The Second Affiliated Hospital of Fujian Medical University, Quanzhou, Fujian, China; ^3^ Department of Pathology, The Second Affiliated Hospital of Fujian Medical University, Quanzhou, Fujian, China

**Keywords:** lung squamous cell carcinoma, chemokine signaling-related genes, signature, prognosis, therapy sensitivity

## Abstract

**Background:** The chemokine signaling pathway plays an essential role in the development, progression, and immune surveillance of lung squamous cell carcinoma (LUSC). Our study aimed to systematically analyze chemokine signaling-related genes (CSRGs) in LUSC patients with stage I–III disease and develop a prediction model to predict the prognosis and therapeutic response.

**Methods:** A total of 610 LUSC patients with stage I–III disease from three independent cohorts were included in our study. Least absolute shrinkage and selection operator (LASSO) and stepwise multivariate Cox regression analyses were used to develop a CSRG-related signature. GSVA and GSEA were performed to identify potential biological pathways. The ESTIMATE algorithm, ssGSEA method, and CIBERSORT analyses were applied to explore the correlation between the CSRG signature and the tumor immune microenvironment. The TCIA database and pRRophetic algorithm were utilized to predict responses to immunochemotherapy and targeted therapy.

**Results:** A signature based on three CSRGs (CCL15, CXCL7, and VAV2) was developed in the TCGA training set and validated in the TCGA testing set and GEO external validation sets. A Kaplan–Meier survival analysis revealed that patients in the high-risk group had significantly shorter survival than those in the low-risk group. A nomogram combined with clinical parameters was established for clinical OS prediction. The calibration and DCA curves confirmed that the prognostic nomogram had good discrimination and accuracy. An immune cell landscape analysis demonstrated that immune score and immune-related functions were abundant in the high-risk group. Interestingly, the proportion of CD8 T-cells was higher in the low-risk group than in the high-risk group. Immunotherapy response prediction indicated that patients in the high-risk group had a better response to CTLA-4 inhibitors. We also found that patients in the low-risk group were more sensitive to first-line chemotherapeutic treatment and EGFR tyrosine kinase inhibitors. In addition, the expression of genes in the CSRG signature was validated by qRT‒PCR in clinical tumor specimens.

**Conclusion:** In the present study, we developed a CSRG-related signature that could predict the prognosis and sensitivity to immunochemotherapy and targeted therapy in LUSC patients with stage I–III disease. Our study provides an insight into the multifaceted role of the chemokine signaling pathway in LUSC and may help clinicians implement optimal individualized treatment for patients.

## Introduction

Lung squamous cell carcinoma (LUSC) is a common type of non-small cell lung cancer (NSCLC) and accounts for approximately 30% of all lung cancers (Nicholson et al., 2022). Most tumors are located in the central part of the lung, usually in the main bronchi, which join the trachea to the lung (Yang et al., 2020). LUSC is associated with adverse clinical outcomes compared to lung adenocarcinoma (LUAD) (Conti and Gatt, 2018). Although striking progress has been made in the past 10 years, including prevention, early detection, targeted therapy, and immunotherapy, the clinical outcome of LUSC remains unsatisfactory (Nasim et al., 2019). The 5-year survival rate for patients with early-stage LUSC is approximately 40%, and the survival rate decreases to just 5% once the cancer is diagnosed in an advanced stage (An et al., 2020). Patients diagnosed with stage I–III LUSC are considered surgically resectable and treated with a combination of surgery or perioperative therapy, including radiotherapy, chemotherapy, and immunotherapy. Compared with surgery alone, perioperative therapy could improve the prognosis for stage I–III LUSC patients. However, not all patients benefit from perioperative therapy. Currently, research efforts are underway to improve the treatment of stage I–III LUSC using precision predictive biomarkers and therapeutic targets. Therefore, effective stratification and prediction of therapeutic response and prognosis will help guide treatment strategies.

Chemokines are a subset of chemoattractant proteins that can specifically induce cell polarization, migration, and immune and inflammatory responses (Zlotnik and Yoshie, 2012). The chemokine–receptor network currently consists of approximately 47 chemokine ligands and 19 seven transmembrane spanning signaling receptors (Zlotnik and Yoshie, 2012). Chemokines are essential for recruitment and activation of cellular migration and have a great impact on tumor progression and metastasis (Cheng et al., 2016a). Recently, chemokines have been increasingly recognized for their pivotal role in regulating the migration and differentiation of immune cells in the tumor microenvironment (TME) (Nagarsheth et al., 2017). For example, Groom et al. reported that CXCL9/CXCL10 and their receptors are significantly associated with Th1-cell responses (Groom et al., 2012). Alicia et al. found that the CCL3/CCL4/CCL5–CCR5 axis induces T-cell antitumor responses by regulating CD8 T-cell activation (González-Martín et al., 2011). However, chemokines also act on tumor cells, modulating their stem-like cell properties, invasiveness, and fibrogenesis (Ozga et al., 2021). These studies suggest that chemokines can serve as prognostic biomarkers and therapeutic targets for cancer. Ryuma et al. reported that a 12-chemokine signature may serve as a predictive indicator for tumor recurrence and host immune status in colorectal cancer patients (Tokunaga et al., 2020). Tao et al. found that a chemokine-based signature could be used to predict the clinical outcome and immunotherapy response in lung adenocarcinoma (Fan et al., 2021). However, few studies have systematically analyzed the relationship between the chemokine signaling pathway and its association with the LUSC immune landscape, therapeutic efficacy, and prognosis.

Chemokine signaling pathways are being widely reported for their chemotactic functions and recruitment of primed effector T-cells into tumors (Rivas-Fuentes et al., 2015). Recently, few studies have systematically analyzed the relationship between chemokine signaling pathways and their association with the immune landscape and prognosis of LUSC. Ma et al. reported that CCL8 rs3138035 may be a candidate predictor for NSCLC survival in Chinese patients (Ma et al., 2011). Artjoms et al. found that statistically significant CXC chemokine concentration changes were positively associated with poor prognosis in NSCLC patients (Spaks, 2017). However, the role of chemokine signaling pathways in the prognosis and therapeutic response of LUSC remains largely unknown. In this study, we sought to investigate the association of chemokine signaling-related biomarker networks with the prognosis of LUSC patients. We attempted to construct a predictive model to predict survival and the response to therapy in patients with early-stage LUSC, which could provide meaningful clues for optimizing an effective treatment for LUSC patients, especially for immunotherapy. Here, we explored the transcriptional profiles, clinical importance, and corresponding pathways of chemokine signaling-related genes (CSRGs) in stage I–III LUSC patients. We developed and validated a prognostic CSRG signature based on three genes. A prognostic nomogram was also constructed to predict the individualized survival probability of LUSC patients. In addition, we further analyzed the signature-related immune landscape and evaluated the practicability of the signature in predicting immunotherapy, chemotherapy, and target therapy sensitivity.

## Materials and methods

### Data collection and preprocessing

The RNA-seq expression data, tumor somatic mutations, and corresponding clinical information of 483 LUSC patients with stage I–III disease were downloaded from The Cancer Genome Atlas (TCGA) database. Patients in the TCGA cohort were randomly divided into the training set (*n* = 339) and the internal testing set (*n* = 144) in a 7:3 ratio. The gene expression profile from TCGA was normalized by the log2 (FPKM+1) formula and then processed by the “limma” R package for differential expression analysis. Representative Gene Expression Omnibus (GEO) datasets containing more than 50 samples from LUSC patients with stage I–III disease were retrieved from the NCBI, including RNA expression data and clinical information. Two independent cohorts, GSE37745 (Botling et al., 2013) (*n* = 66) and GSE30219 (Rousseaux et al., 2013) (*n* = 61), which were based on the GPL570-55999 Array platform, were used as external validation sets. A total of 185 chemokine signaling-related genes (CSRGs) were obtained from the Kyoto Encyclopedia of Genes and Genomes (KEGG) database (https://www.genome.jp/kegg) (Supplementary Table S1). In addition, tumor stemness scores based on mRNA (RNAss) and DNA methylation (DNAss) were downloaded from the UCSC Xena database (https://xenabrowser.net/datapages/).

### Construction of the chemokine signaling-related gene signature

A univariate Cox regression analysis was used to screen the prognostic genes in the training set. Least absolute shrinkage and selection operator (LASSO) regression analysis was utilized to minimize the risk of overfitting and identify the most important genes. A stepwise Cox regression model was then developed using multivariate Cox analysis: risk score = coef 1 × exp^gene1^ + coef 2 × exp^gene2^……coef n × exp^gene n^. All LUSC patients were divided into a high-risk group and a low-risk group based on the median risk score. The Kaplan–Meier method and the log-rank test were used to analyze survival. A time-dependent receiver operating characteristic (ROC) curve analysis was used to evaluate the performance of the risk scores. Decision curve analysis (DCA) was performed to verify the predictive value of the nomogram compared to that of other independent factors (Fitzgerald et al., 2015).

### Functional enrichment analysis

Gene set variation analysis (GSVA) is a nonparametric, unsupervised method based on a list of functional terms or gene sets that allows pathway enrichment to be evaluated for each sample. We used the “GSVA” R package to evaluate the differences in functional pathways between the high-risk and low-risk groups. The Kyoto Encyclopedia of Genes and Genomes (KEGG) pathway gene set “c2. cp.kegg.v7.5.1. symbols” was downloaded from the MSigDB database for the GSVA using the “clusterProfiler” and “GSVA” R packages. An adjusted *p* < 0.05 was considered to indicate a statistically significant difference in pathways between the groups (Hanzelmann et al., 2013; Kanehisa et al., 2017).

Gene set enrichment analysis (GSEA) is a computational method belonging to functional class scoring approaches that identifies whether a preselected set of genes is differentially expressed between the groups. In this study, GSEA was used to investigate differential biological functions between the high-risk and low-risk groups based on the gene set “c5. go.v7.5.1. symbols.gmt.” Gene Ontology (GO) enrichment analysis, including biological processes (BP), molecular functions (MF), and cellular components (CC), was conducted using the “clusterProfiler” and “org.Hs.e.g.db” R packages (Ashburner et al., 2000).

### Evaluation of immune cell infiltration in the tumor microenvironment

“ESTIMATE” is a method for predicting tumor purity and the fraction of infiltrating stromal/immune cells in tumor samples using gene expression data. Its algorithm is based on single-sample Gene Set Enrichment Analysis (ssGSEA). The “ESTIMATE” algorithm was used to calculate the immune score, stromal score, and tumor purity of each sample using the “estimate” R package (Yoshihara et al., 2013).

ssGSEA is an extension of gene set enrichment analysis (GSEA) that provides a separate enrichment score for each sample and gene set. The ssGSEA score represents the degree to which the genes in a specific gene set are coordinately upregulated or downregulated within a sample. In this study, we analyzed the expression of 29 immunity-related signatures that represented different immune cell functions and pathways using the ssGSEA algorithm in the “GSEAbase” and “GSVA” R packages (Bindea et al., 2013).

CIBERSORT is an analytical tool used to assess the abundances of a particular cell type in a mixed cell population based on gene expression profiles. The CIBERSORT algorithm in the “CIBERSORT” R package was used to estimate the relative abundances of 22 types of immune cells in each sample based on the gene expression data of LUSC patients, and results with a *p* value ≤0.05 were eligible for further analysis (Newman et al., 2015; Chen et al., 2018).

### Immunotherapy response prediction and drug sensitivity analysis

The immunophenotype score (IPS) is a measure of the whole immunogenicity of an individual solid tumor, with a higher score representing a better response to immunotherapy (Hugo et al., 2016). The IPS, which ranges from 0 to 10, was calculated according to the expression of representative gene sets. The IPSs of LUSC patients were acquired from The Cancer Immunome Atlas (TCIA) (https://tcia.at/home) database, which uses machine learning to build a scoring scheme for the quantification termed IPS. The IPSs of CTLA-4 and PD-1 blockers were used to predict the immunotherapy efficacy of the patient response to anti-CTLA-4 and anti-PD-1 antibodies.

The pRRophetic algorithm is a tool to predict the clinical chemotherapeutic and target therapy sensitivity using tumor gene expression data. We used the pRRophetic algorithm to estimate the therapeutic compound response based on the half-maximal inhibitory concentration (IC_50_) of each LUSC sample, which was based on gene expression and drug sensitivity data from cell lines in the Cancer Genome Project (CGP) (Geeleher et al., 2014).

### Clinical specimen collection and quantitative real-time PCR analysis

A total of 10 pairs of matched LUSC cancer tissues and adjacent normal tissues were collected from The Second Affiliated Hospital of Fujian Medical University. This study was approved by the Ethics Committee of The Second Affiliated Hospital of Fujian Medical University.

For tissue total RNA isolation, 1 ml of TRIzol (Invitrogen, United States) was added to 50–100 mg of tissue, and the total RNA samples were extracted following the manufacturer’s protocol. The RNA was then reverse-transcribed using a cDNA Synthesis Kit (TaKaRa, Japan). Real-time PCR was performed on the RT‒PCR System using SYBR Premix Ex Taq (TaKaRa). The relative CCL15 and PPBP mRNA expression levels were normalized to those of GAPDH. The relative expression was calculated with the 2^−ΔΔ^CT method. The PCR primers were synthesized by Sangon Biotech (Shanghai, China) and are listed in Supplementary Table S2.

## Results

### Development of a prognostic model based on chemokine signaling-related genes in stage I–III lung squamous cell carcinoma patients

The detailed design of this study is illustrated in Supplementary Figure S1. The list of R packages used in this study is shown in Supplementary Table S3.

**FIGURE 1:**
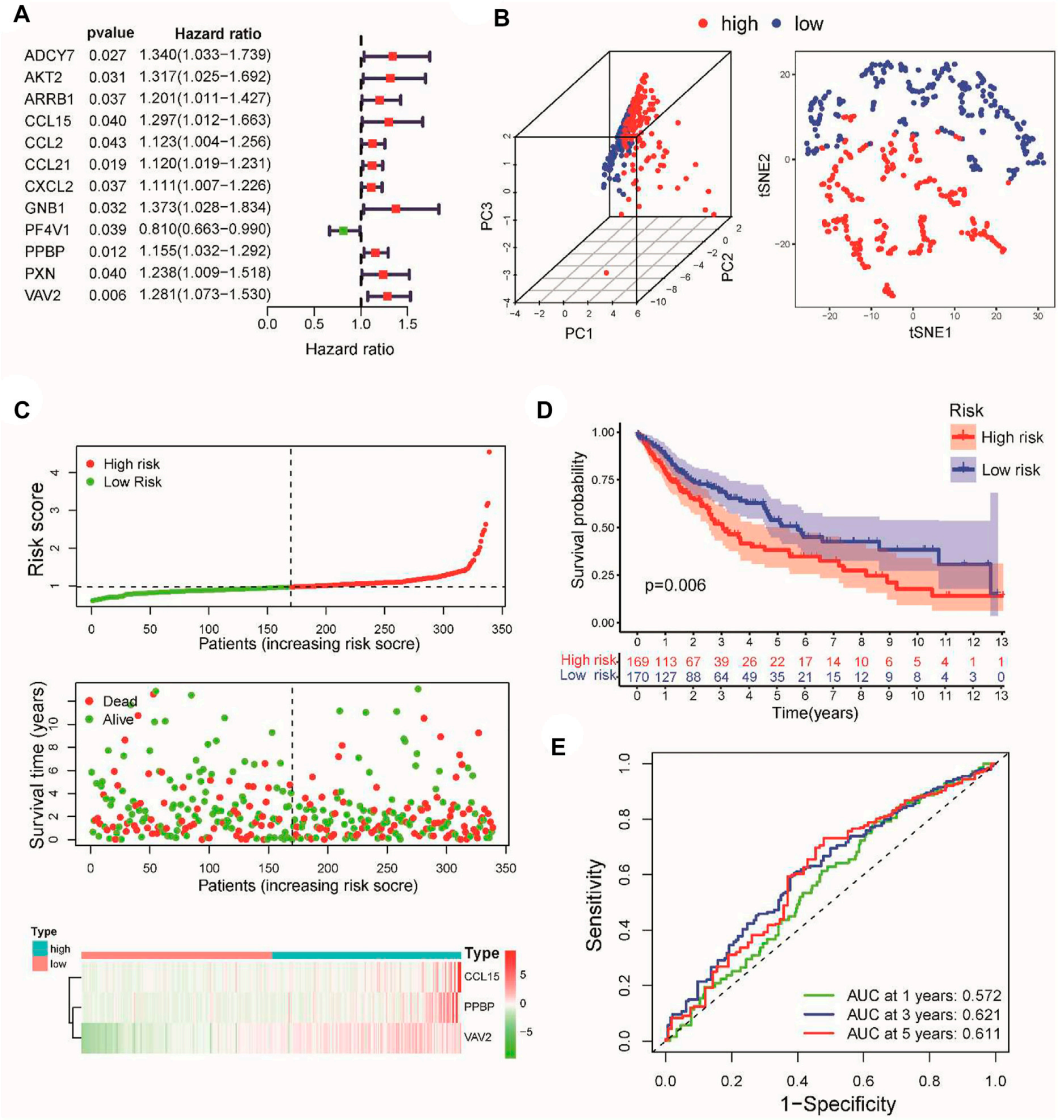
Construction of a prognostic model for stage I–III LUSC patients according to three CSRGs. **(A)** Identifying 12 prognostic CSRGs using univariate Cox regression analysis. **(B)** Principal component analysis (PCA) based on the expression levels of three CSRGs in the TCGA training set. **(C)** Distribution of risk scores, survival status, and three CSRG expression panels in the training set. **(D)** Kaplan–Meier survival analysis of OS between the high-risk and low-risk groups. **(E)** Time-dependent ROC for 1-, 3-, and 5-year OS predictions for the CSRG prognostic signature in the training set.

Patients in the TCGA cohort were randomly divided into the training set (*n* = 339) and the internal testing set (*n* = 144) in a 7:3 ratio. The clinical information did not significantly differ between the two sets (Table 1). A total of 25 upregulated and 11 downregulated differentially expressed CSRGs (|log2-fold change| >1, FDR< 0.05) were identified between tumor and normal tissues based on TCGA training cohorts (Supplementary Figure S2A). As screened by univariate Cox regression, 12 CSRGs were statistically significantly associated with the overall survival (OS) of LUSC patients (Figure 1A). Among these genes, 11 were risk factors, and only one was a protective factor in LUSC. A LASSO regression analysis was utilized to minimize the risk of overfitting (Supplementary Figure S2B). Subsequently, a prognostic signature consisting of three CSRGs was developed *via* a stepwise multivariate Cox regression analysis. According to the regression coefficients and expression levels of the three CSRGs, the risk score of each sample was calculated as follows: risk score = (0.2618 ×  mRNA level of CCL15) + (0.1277 ×  mRNA level of CXCL7(PPBP)) + (0.1813 ×  mRNA level of VAV2). Based on the optimal cutoff value of the risk score, patients with stage I–III LUSC were divided into high-risk and low-risk groups. Principal component analysis (PCA) and t-distributed stochastic neighbor embedding (tSNE) based on the expression levels of CSRGs showed a significant distribution difference between the high-risk and low-risk groups (Figure 1B). As the risk score increased, the patients in the high-risk group had a higher probability of death than those in the low-risk group (Figure 1C). A Kaplan–Meier survival analysis revealed that patients in the high-risk group had significantly shorter OS than those in the low-risk group (Figure 1D). The time-dependent ROC curve showed that the AUC values for the 3- and 5-year OS were 0.62 and 0.61, respectively (Figure 1E). These results supported the relatively good sensitivity and accuracy of our prognostic signature.

**TABLE 1:** Clinicopathological characteristics of LUSC patients from TCGA and GEO cohorts.

Characteristics	TCGA training set (*n* = 339)	TCGA testing set (*n* = 144)	GSE37745 (*n* = 66)	GSE30219 (*n* = 61)
TNM stage				
Stage I	178 (52.51%)	64 (44.44%)	40 (60.6%)	—
Stage II	111 (32.74%)	47 (32.64%)	15 (22.73%)	—
Stage III	50 (14.75%)	33 (22.92%)	11 (16.67%)	—
Pathologic N				
N0	221 (65.19%)	89 (61.81%)	—	—
N1	89 (26.25%)	35 (24.31%)	—	—
N2	21 (6.19%)	18 (12.5%)	—	—
N3	4 (1.18%)	1 (0.69%)	—	—
Nx	4 (1.18%)	1 (0.69%)	—	—
Pathologic T				
T1	84 (24.78%)	30 (20.83%)	—	49 (80.32%)
T2	195 (57.52%)	86 (59.72%)	—	6 (9.84%)
T3	46 (13.57%)	22 (15.28%)	—	4 (6.56%)
T4	14 (4.13%)	6 (4.17%)	—	2 (3.28%)
Age				
≤65	127 (37.47%)	60 (41.67%)	29 (43.94%)	36 (59.02%)
>65	212 (62.53%)	84 (58.33%)	37 (56.06%)	25 (40.98%)
Gender				
Female	86 (25.37%)	41 (28.47%)	20 (30.3%)	5 (8.2%)
Male	253 (74.63%)	103 (71.53%)	46 (69.7%)	56 (91.8%)
Smoking history				
No	48 (14.16%)	25 (17.36%)	—	—
Yes	291 (85.84%)	119 (82.64%)	—	—

### Internal and external validation of the chemokine signaling-related gene prognostic signature

**FIGURE 2:**
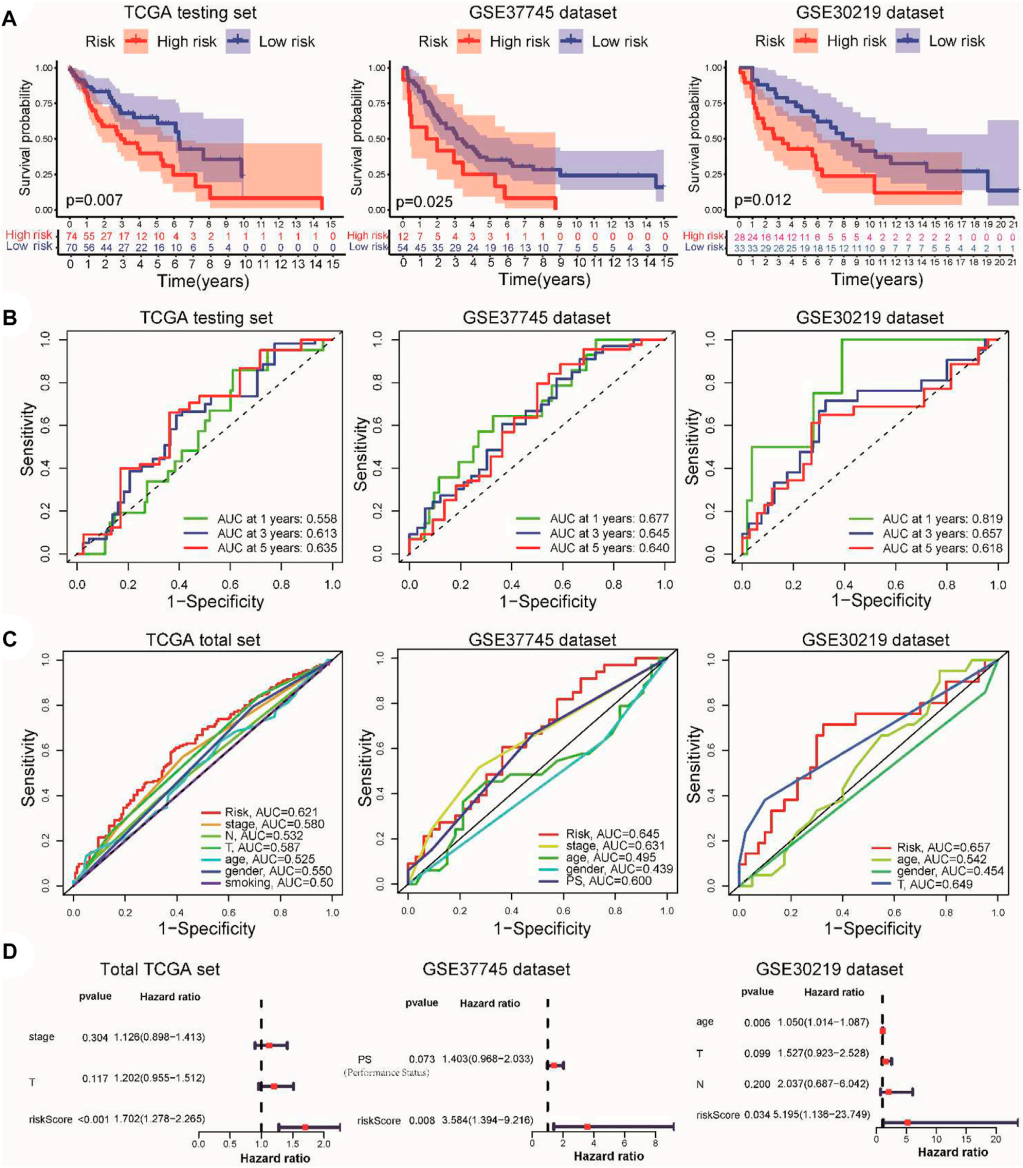
Validation of the prognostic signature in TCGA internal testing and GEO external datasets. **(A)** Kaplan–Meier survival analysis of overall survival between the high- and low-risk groups in the validation datasets. **(B)** Time-dependent ROC for 1-, 3-, and 5-year OS predictions for the prognostic signature in validation datasets. **(C)** Time-dependent ROC curves for clinical characteristics and risk score for 3-year OS of LUSC in TCGA set and GEO datasets. **(D)** Multivariate Cox regression analyses of the risk score in the total TCGA cohort and GSE37745 and GSE30219 external validation cohorts.

To verify the predictive power of our prognostic signature, the testing set and the GSE37745 and GSE30219 datasets served as the validation cohorts. Using the same calculation formula of the risk score, patients with stage I–III LUSC were assigned into high-risk and low-risk groups according to the median value of the risk score from the training set. As expected, the distributions of risk scores, survival status, and CSRG expression were consistent with these results in the training set (Supplementary Figure S3A). The verification results demonstrated that patients in the low-risk group exhibited a better prognosis than those in the high-risk group (Figure 2A). In the internal testing set, the AUCs of the risk signature for predicting the 1-, 3-, and 5-year OS were 0.558, 0.613, and 0.635, respectively. For the external validation datasets, the AUC values in the GSE37745 and GSE30219 datasets exceeded 0.6 (Figure 2B). The time-dependent ROC curve involving various clinical characteristics and risk scores for 3-year survival demonstrated that the CSRG signature had better predictive efficiency than other clinical factors (Figure 2C). Furthermore, we applied univariate and multivariate Cox regression analyses to examine the independent prognostic value of this signature for LUSC in TCGA and validation cohorts. Univariate (Supplementary Figure S3B) and multivariate (Figure 2D) Cox regression models demonstrated that this signature could serve as an independent predictor for LUSC patients in TCGA and validation cohorts. Furthermore, we compared the performance of the present signature with those of the other previously reported gene signatures in LUSC. The RMS curve showed that the performance of the present signature was similar to that of the other four signatures (Supplementary Figure S3C) (Huang et al., 2021; Li et al., 2021; Li et al., 2022; Zhai et al., 2022). These results suggest that the CSRG signature can serve as a candidate prognostic biomarker for survival in patients with stage I–III LUSC.

### Establishment of a nomogram for predicting the prognosis of lung squamous cell carcinoma patients with stage I–III

**FIGURE 3:**
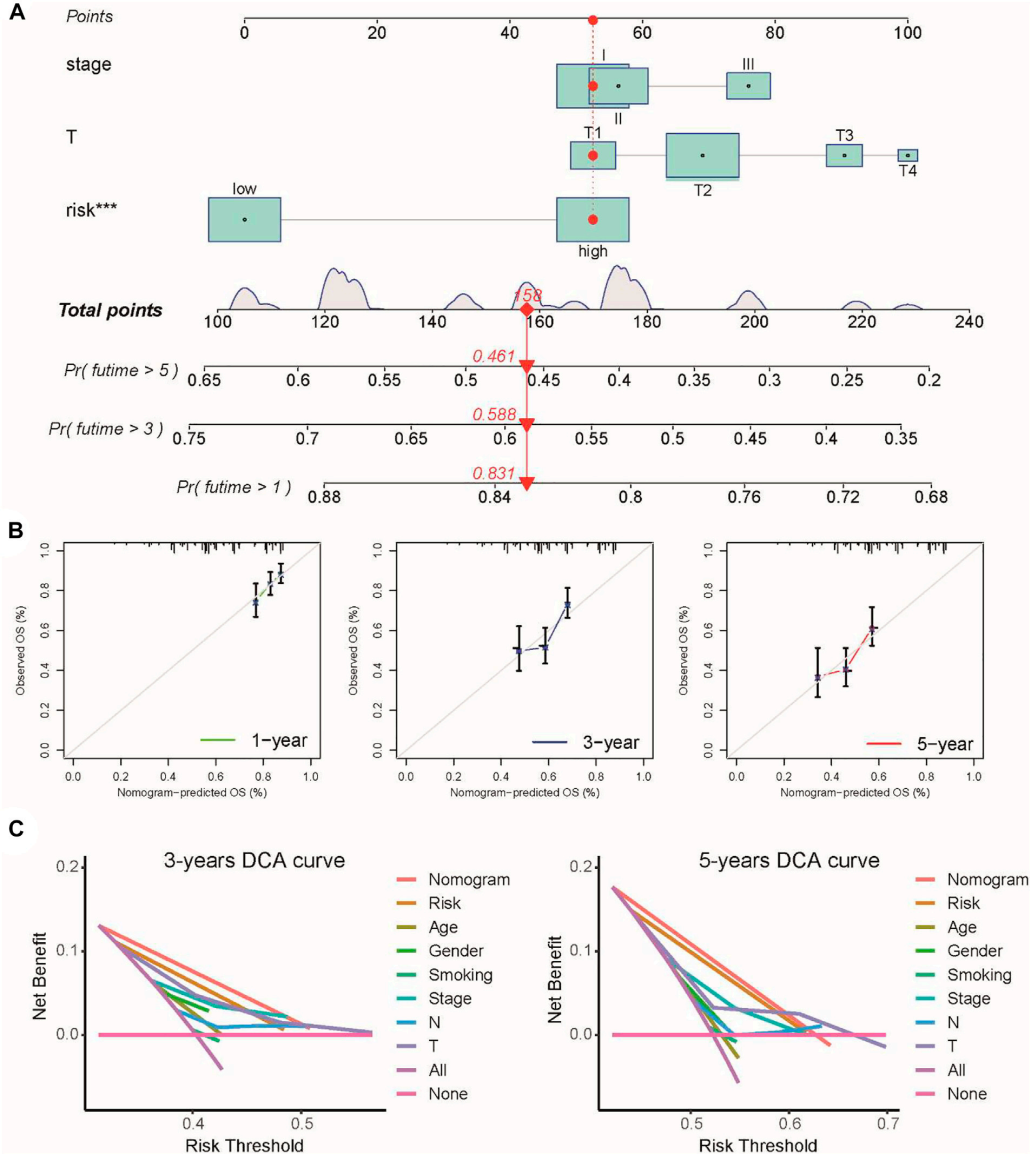
Prognostic nomogram was constructed by combining clinical stage, T stage, and risk score in the TCGA cohort. **(A)** Prognostic nomogram for predicting the 1-, 3-, and 5-year survival rates of LUSC patients. **(B)** Corresponding calibration curve for 1-, 3-, and 5-year OS prediction. **(C)** DCA curve for the prediction of 3-year and 5-year overall survival.

To further extend the clinical applicability of the prognostic signature, we constructed a prognostic nomogram combined with clinical stage, T stage, and risk score to predict the 1-, 3-, and 5-year OS of LUSC patients in the TCGA cohort (Figure 3A). The calibration curves of this prognostic nomogram showed that our nomogram had the ability to accurately predict the actual 1-, 3-, and 5-year survival rates (Figure 3B **)**. In addition, decision curve analysis (DCA) was used to compare the net benefits of different factors, including none, all, risk score, and nomogram. DCA curves also demonstrated that the prognostic nomogram had better predictive capability than the TNM staging system (Figure 3C). These results indicated that the prognostic nomogram has good discrimination and accuracy and can be applied to assess the prognosis of LUSC patients with stage I–III disease.

### Clinical correlation and functional evaluation of the chemokine signaling-related gene signature

**FIGURE 4:**
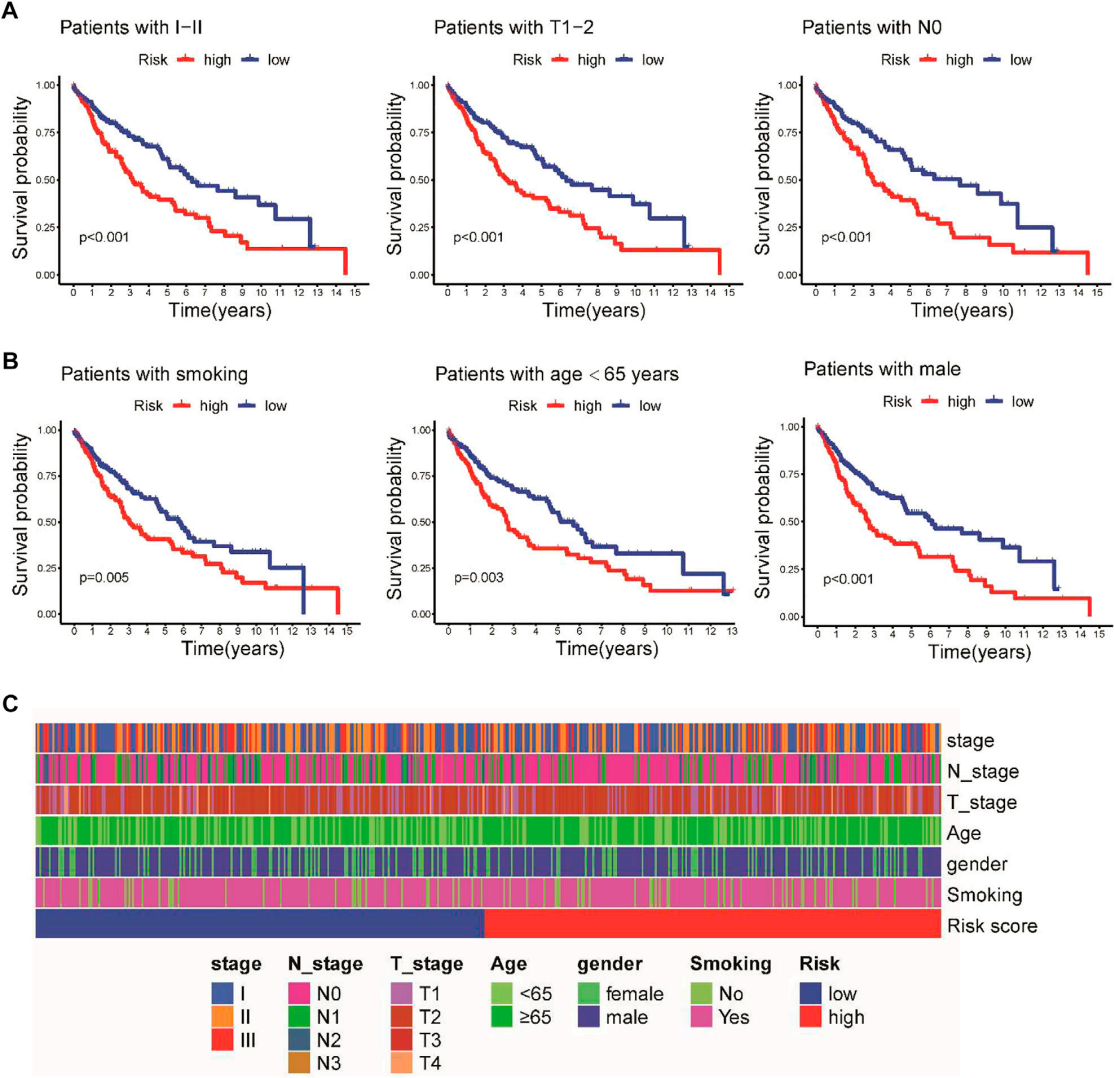
Stratified Kaplan–Meier survival analysis of different clinical subgroups in the TCGA cohort. **(A)** Clinical I-II stage, T1-2, and N0 pathologic stage. **(B)** Smoking status, age, and sex. **(C)** Heatmap illustrating the associations between the risk score and clinicopathological characteristics.

To further investigate the prognostic effect of this model in clinical practice, we evaluated the association between the prognostic signature and different clinical subgroups in the TCGA cohort. A Kaplan–Meier survival analysis demonstrated that patients with early-stage disease in the low-risk group, including stage I–II, T1-2, and N0 pathologic stage disease, had a better prognosis (Figure 4A). Moreover, the prognosis of high-risk patients based on smoking status, age, and gender was poorer than that of low-risk patients (Figure 4B). We further analyzed the associations between the risk score and these clinicopathological parameters, but the risk score was not significantly associated with clinicopathological characteristics (Figure 4C).

**FIGURE 5:**
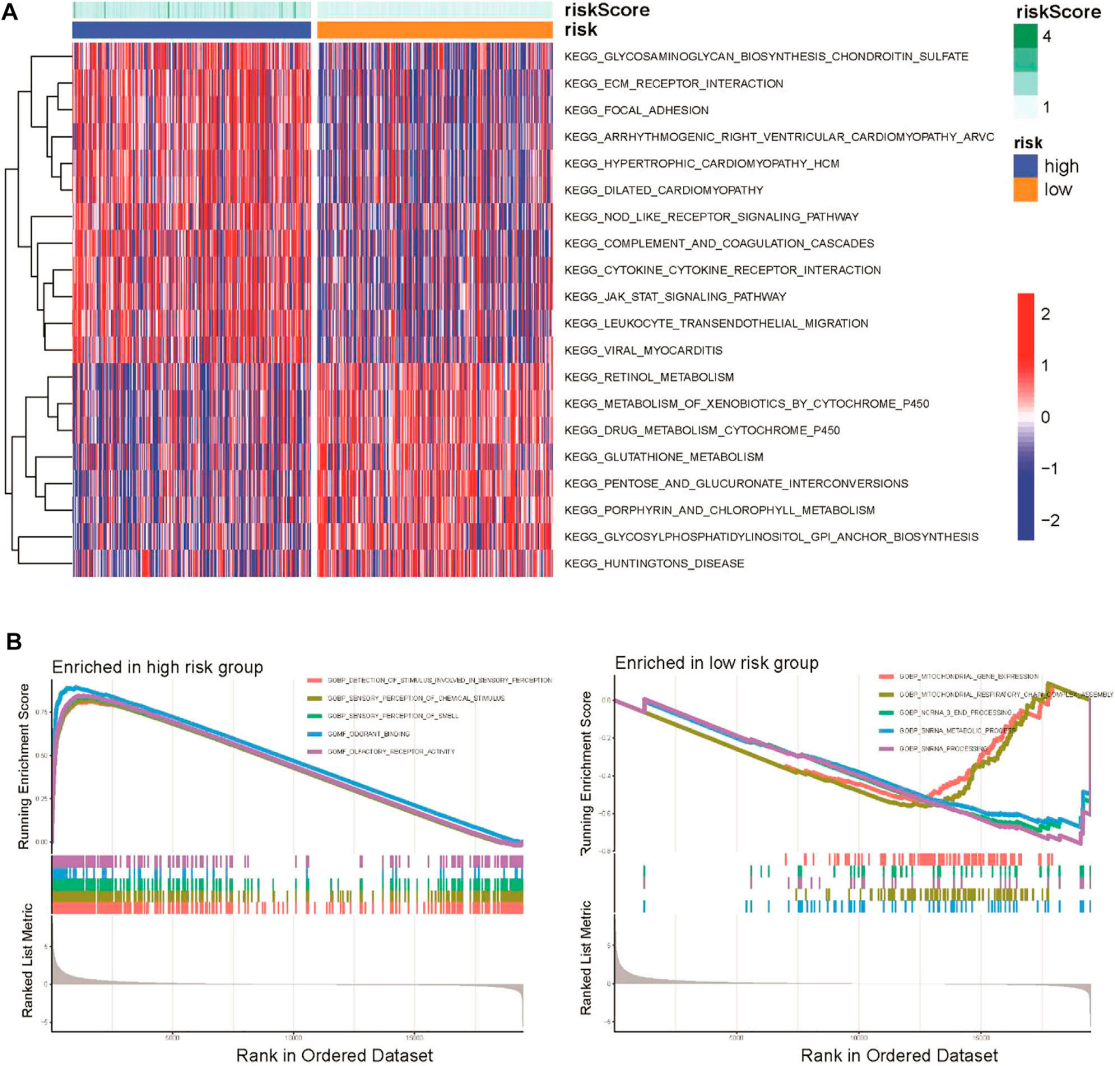
Functional and biological pathway analysis of the CSRG prognostic signature. **(A)** Visualization of pathway enrichment analysis by GSVA between the high-risk and low-risk groups. **(B)** GSEA of biological pathways between the high-risk and low-risk groups.

To explore the potential biological functions of the CSRG signature, GSVA enrichment analysis and GSEA were applied to identify the hub signaling pathways between the high-risk and low-risk groups. GSVA showed that the high-risk group was enriched in ECM–receptor interaction, the Nod-like receptor (NLR) signaling pathway, and the cytokine–cytokine receptor interaction pathway, whereas pentose and glucuronate interconversion, metabolism of xenobiotics cytochromes P450, and drug metabolism cytochromes P450 were enriched in the low-risk group (Figure 5A). The GSEA results indicated that the high-risk group was enriched in stimuli involved in sensory perception, olfactory receptor activation, and the sensory perception of chemical stimuli (Figure 5B).

### Correlation between the chemokine signaling-related gene signature and the tumor immune microenvironment

**FIGURE 6:**
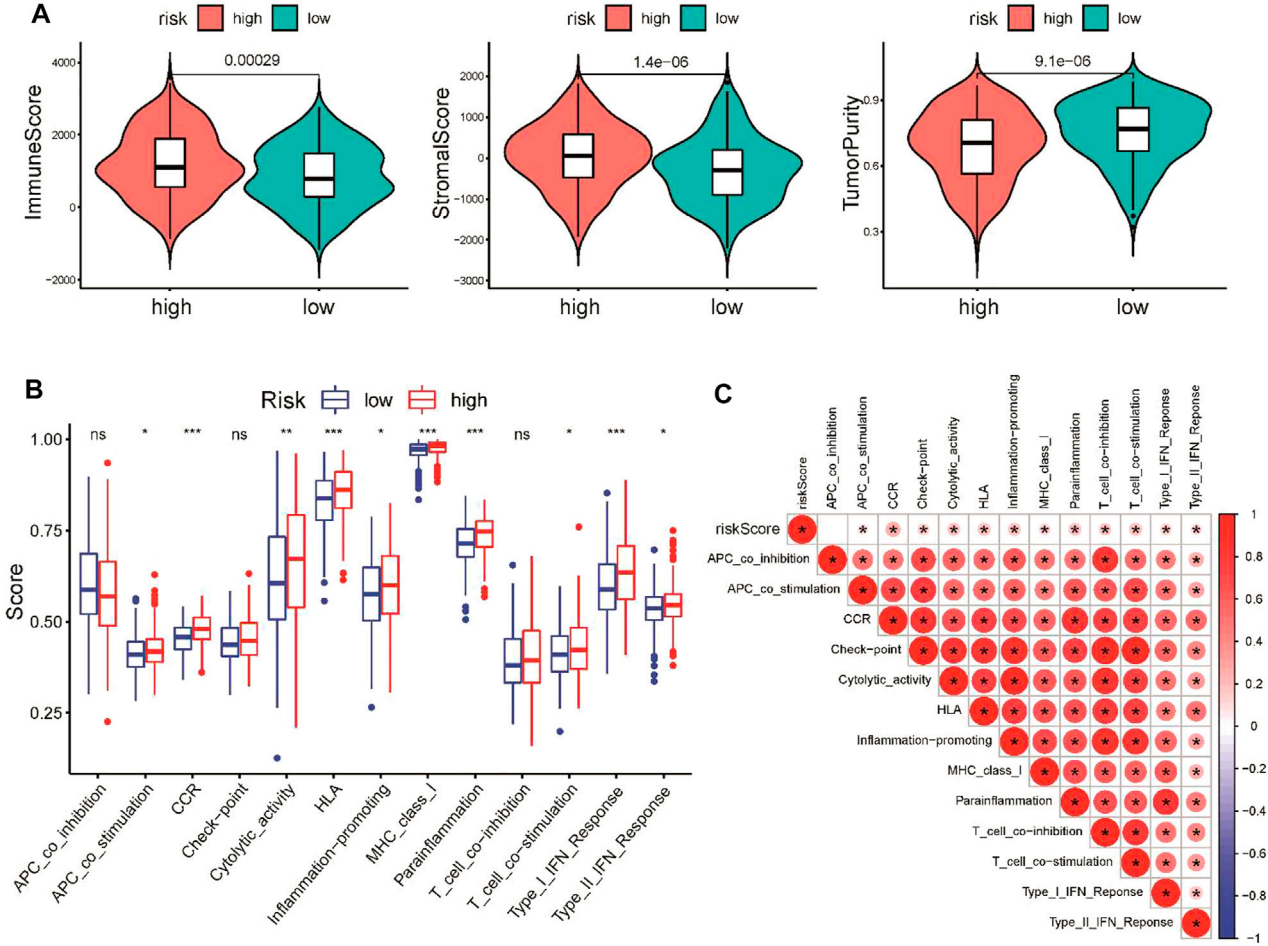
Comparison of immune activity between the high-risk and low-risk groups. **(A)** Comparison of immune score, stromal score, and tumor purity between the low- and high-risk groups. **(B)** Comparison of the enrichment scores of 13 immune-related pathways between the low- and high-risk groups. **(C)** Correlation of the risk score and immune-related pathways. *p < 0.05, **p < 0.01, and ***p < 0.001.

An increasing number of studies have reported that the chemokine signaling pathway plays crucial roles in the tumor microenvironment by mediating various processes (Bule et al., 2021). To further explore the correlation between the CSRG signature and the TME, ESTIMATE algorithm, ssGSEA method, and CIBERSORT analyses were performed to evaluate differences in the immune landscape between the high-risk and low-risk groups in TCGA cohort. The ESTIMATE analysis demonstrated that the immune score and stromal score were increased, while tumor purity was decreased in the high-risk group (Figure 6A). ssGSEA was applied to estimate immune-related functions, and we found that the high-risk group was significantly enriched in several pathways related to the immune response, including type I IFN responses, cytolytic activity, and inflammation promotion (Figure 6B). A correlation analysis showed that the risk score was positively associated with most immune-related pathways (Figure 6C).

**FIGURE 7:**
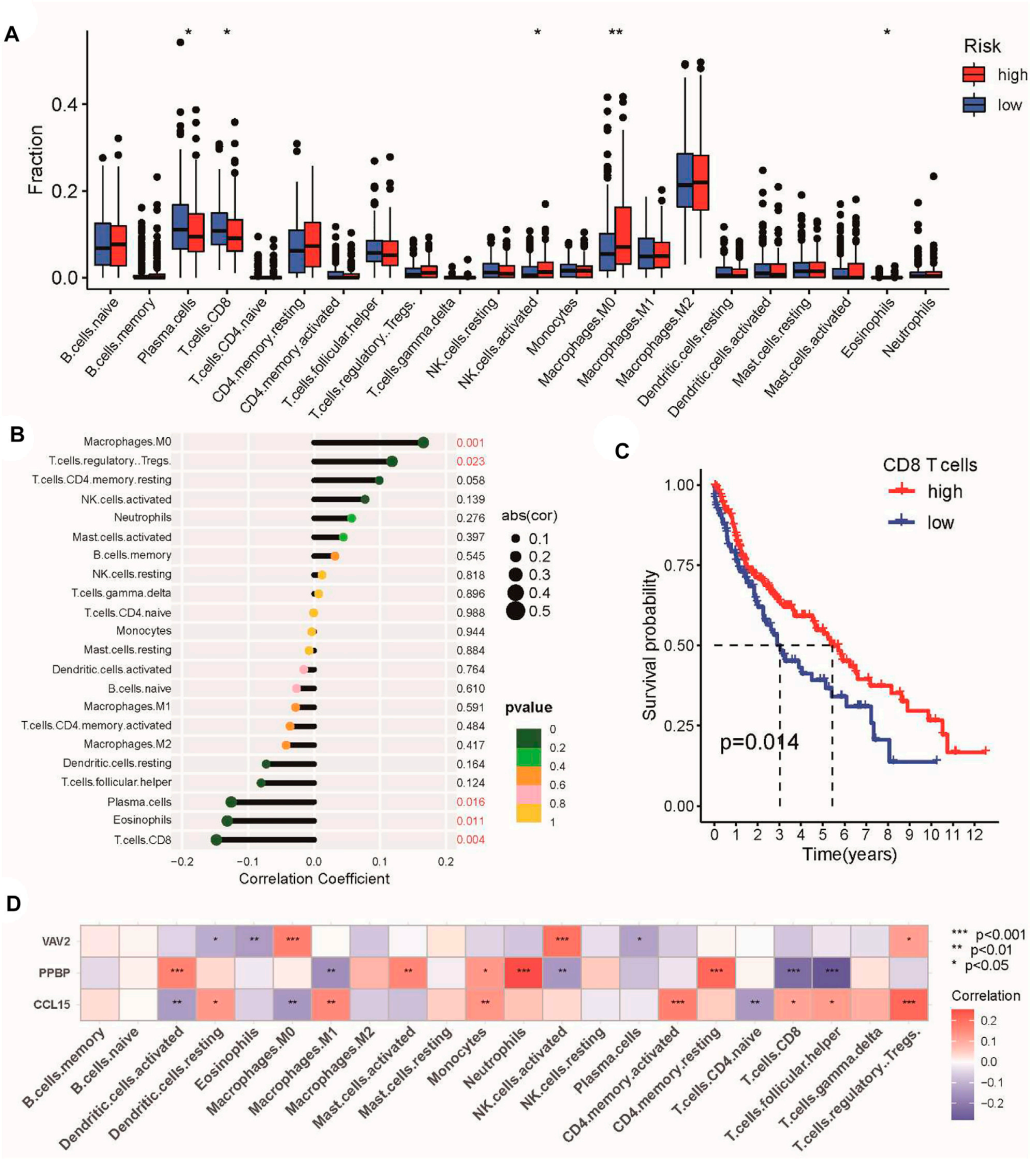
Characteristics of immune infiltrating cells in the high-risk and low-risk groups. **(A)** Differences in the abundance of immune-infiltrating cells between the high-risk and low-risk groups. **(B)** Correlation analysis between immune-infiltrating cells and the risk score. **(C)** Kaplan–Meier analysis of prognosis according to the proportions of CD8 T-cells. **(D)** Correlation analysis of immune cells and three genes in the CSRG signature. *p < 0.05, **p < 0.01, and ***p < 0.001.

Subsequently, CIBERSORT was performed to analyze the proportions of 22 immune-infiltrating cells in the tumor microenvironment. Our results showed that the abundance of CD8 T-cells, plasma cells, and eosinophils was elevated in the low-risk group (Figure 7A), which was negatively associated with the risk score, whereas M0 macrophages and regulatory T-cells were positively correlated with the risk score (Figure 7B). In addition, we found that patients with higher proportions of CD8 T-cells had a better prognosis for OS (Figure 7C). Finally, we examined the correlation between the three genes in the signature and infiltrating immune cells to better understand the molecular mechanism. We found that PPBP (CXCL7) was negatively associated with CD8 T-cells, whereas CCL15 was positively correlated with Treg cells (Figure 7D). Taken together, the abovementioned results all revealed the close relationship between the CSRG signature and tumor immunity in LUSC.

### Association between the chemokine signaling-related gene signature and immunotherapeutic sensitivity in lung squamous cell carcinoma

**FIGURE 8:**
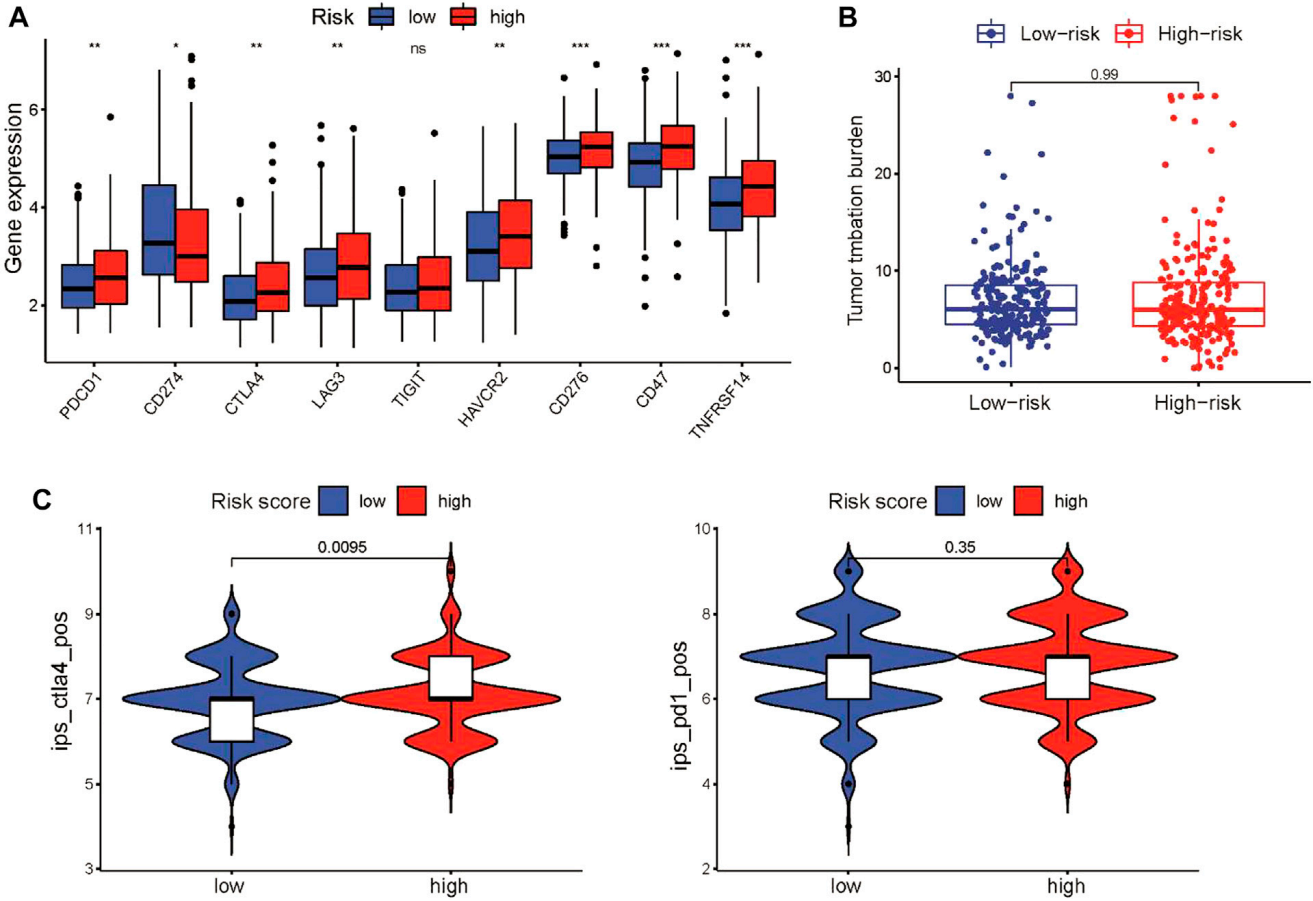
Characteristics of immune infiltrating cells in the high-risk and low-risk groups. **(A)** Box plots showing the relationship between the risk score and the expression level of coinhibitory immune checkpoint genes in the TCGA cohort. **(B)** TMB did not differ between the high-risk and low-risk groups. **(C)** Violin diagram showing the IPSs for CTLA-4 and PD-1 inhibitors for the two groups. *p < 0.05, **p < 0.01, and ***p < 0.001.

The CSRG signature closely correlated with immune activity in the tumor microenvironment, which can affect the response of patients to immunotherapy. Therefore, we investigated the association between the CSRG signature and immunotherapeutic sensitivity in LUSC patients. First, we evaluated the expression of immune checkpoint genes, including PD-1, PD-L1, CTLA4, LAG-3, TIM-3, TIGIT, HVEM, and CD47, between the two groups. We found that the expression of coinhibitory immune checkpoint genes in the high-risk group was higher than that in the low-risk group, except for PD-L1 (Figure 8A). We further examined the correlation between the risk score and TMB, which commonly serves as an immunotherapy biomarker. However, the risk score and TMB did not correlate (Figure 8B). Finally, we used the IPS score to compare the efficacy of immunotherapy for patients in the high-risk and low-risk groups. The IPS of the CTLA-4 inhibitor in the high-risk group was higher than that in the low-risk group, which indicated that patients in the high-risk group had a better response to the CTLA-4 inhibitor, whereas the IPS for the PD-1 inhibitor did not differ between the groups (Figure 8C).

### Chemokine signaling-related gene signature correlated with sensitivity to chemotherapy and targeted therapy

**FIGURE 9:**
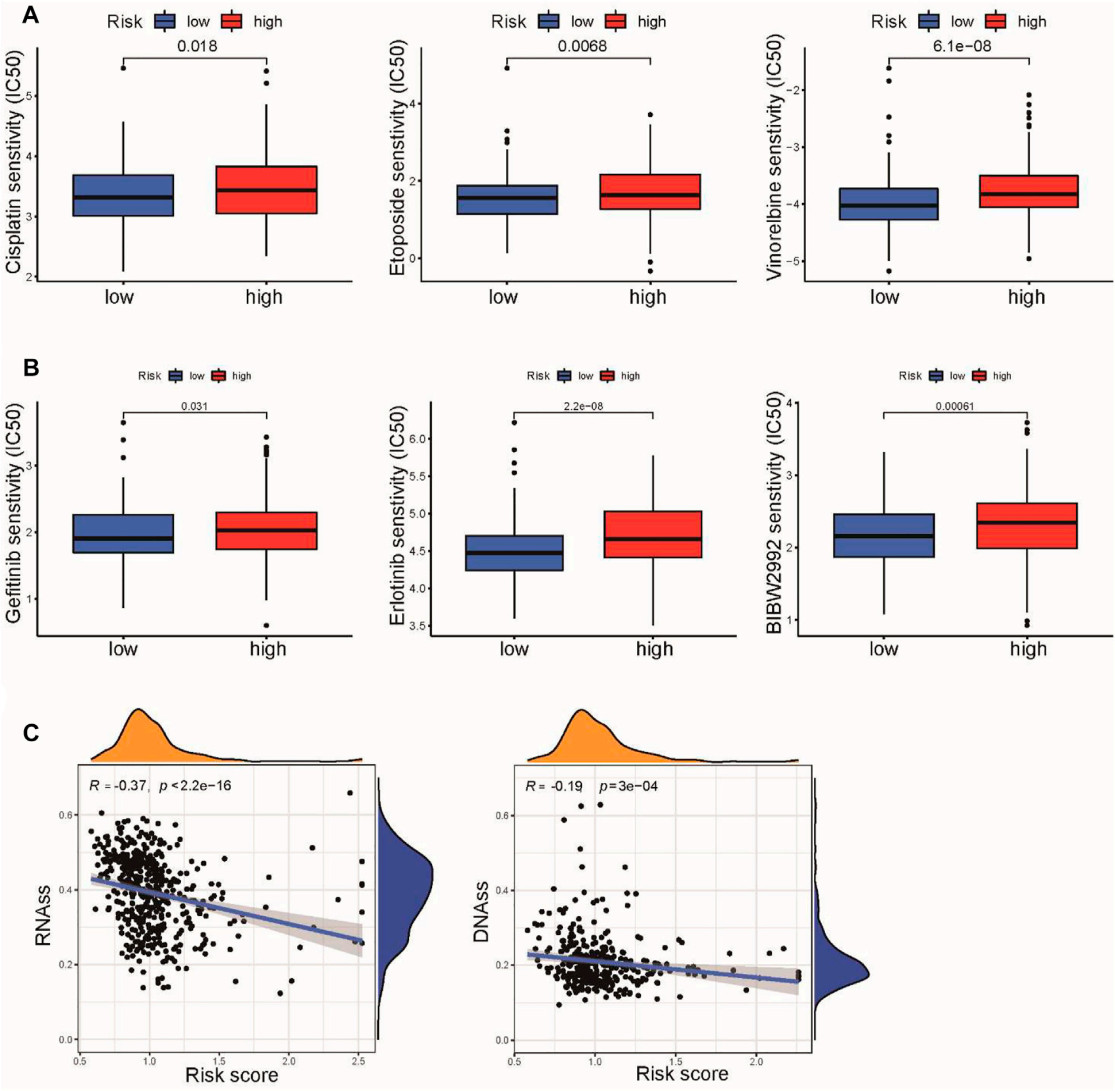
Association of risk score with chemotherapy and target therapy sensitivity in LUSC. **(A)** IC_50_ values of three chemotherapeutic drugs, cisplatin, etoposide, and vinorelbine, were calculated based on the pRRophetic algorithm. **(B)** IC_50_ values of three EGFR inhibitors, gefitinib, erlotinib, and afatinib, were calculated based on the pRRophetic algorithm. **(C)** Correlation between the risk score and cancer stemness scores of RNAss and DNAss.

We next evaluated the practicability of the signature in predicting the response to chemotherapy and targeted therapy for LUSC patients. Based on the pRRophetic algorithm, our results revealed that three common chemotherapeutic drugs for LUSC (cisplatin, etoposide, and vinorelbine) had higher IC_50_ values in high-risk patients, indicating that low-risk patients were more sensitive to these three drugs (Figure 9A). We also found that the IC_50_ values of several EGFR inhibitors (gefitinib, erlotinib, and afatinib) were increased in the high-risk group compared to the low-risk group (Figure 9B). Chemokine signaling pathways reportedly play multifaceted roles in tumor biology, including regulating tumor stemness, which is associated with chemotherapy response (Nagarsheth et al., 2017). We further explored the correlation between the risk score and tumor stemness in TCGA cohort. We observed that the risk score was negatively associated with RNAss and DNAss in LUSC (Figure 9C). These results suggested that the CSRG signature can be used as a potential predictor of chemical sensitivity for LUSC patients.

### Validation of the expression of core genes in the chemokine signaling-related gene signature

**FIGURE 10:**
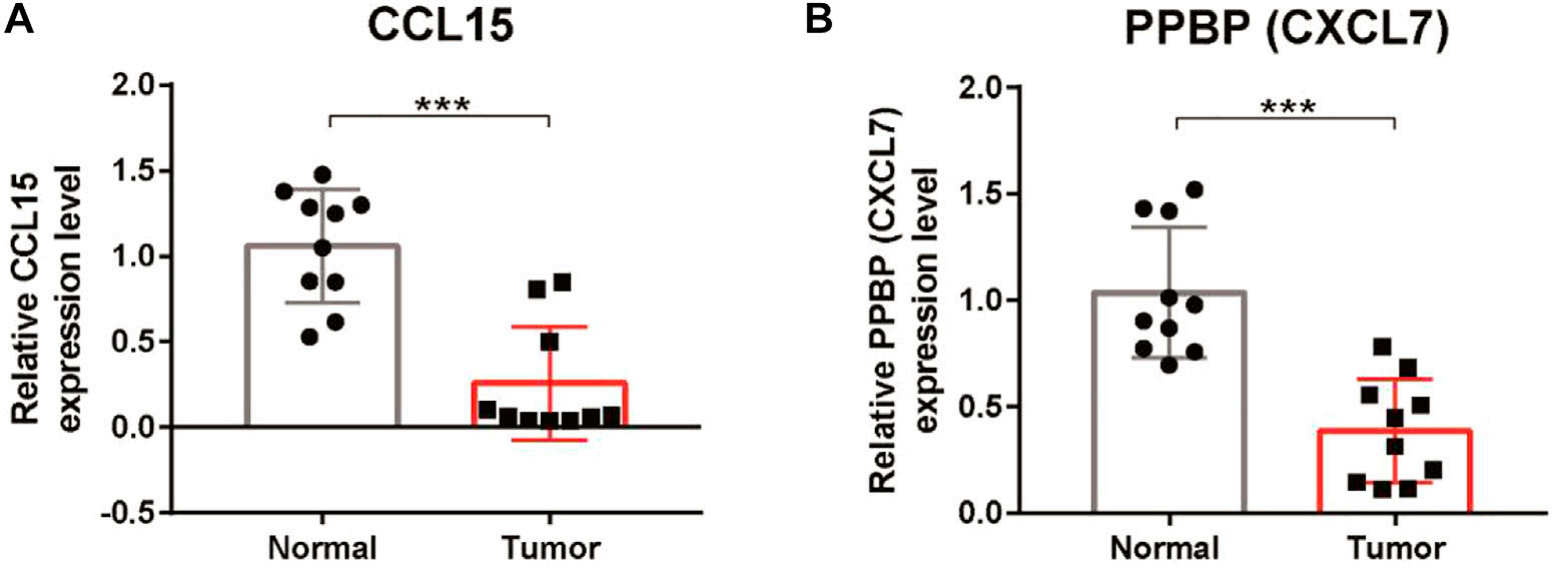
Verification of the expression of CCL15 and PPBP in LUSC tissues. **(A)** qRT‒PCR analysis of the relative mRNA levels of CCL15 in tumor tissues compared with adjacent normal tissues. **(B)** qRT‒PCR analysis of the relative mRNA levels of PPBP in tumor tissues compared with adjacent normal tissues. *p < 0.05, **p < 0.01, and ***p < 0.001.

To further verify the results of the bioinformatic analysis, the expression levels of CCL15 and PPBP were validated by qRT‒PCR in clinical tumor specimens. We observed that the expression levels of CCL15 and PPBP were downregulated in tumor tissues compared with the adjacent normal tissues (Figures 10A,B). These results were consistent with those of our bioinformatics analysis, which suggested that our signature was a reliable prognostic model for LUSC patients.

## Discussion

Over the past 10 years, significant progress has been made in the diagnosis and treatment of lung cancer, especially in immunotherapy and targeted therapy (Jabbour et al., 2022; Reck et al., 2022). Unfortunately, compared with LUAC, targeted treatments for LUSC patients remain unsuccessful (Chang et al., 2021). Moreover, immunotherapeutic approaches have revolutionized the treatment of advanced LUSC (Gettinger et al., 2021; Mielgo-Rubio et al., 2021), and chemotherapy combinations have become the first-line treatment for advanced LUSC (Karachaliou et al., 2018). To further improve the prognosis and therapeutic strategies for LUSC, an in-depth understanding of the mechanisms underlying immune evasion is critical for accurate identification of predictive biomarkers for patient stratification and development of effective therapies in LUSC patients. Considering that the chemokine signaling pathway plays a critical role in the initiation of the tumor immune response of LUSC, the development of a predictive signature based on chemokine signaling-related genes will be meaningful for therapeutic decision-making.

Recently, a growing body of evidence has shown that chemokines also play an important role in various tumor-related processes (Korbecki et al., 2020). Studies have reported that several chemokines, such as CCL2, CCL5, CCL4, CXCL19, and CXCL12, are associated with the progression, metastasis, and prognosis of lung cancer (Wagner et al., 2009; Itakura et al., 2013; Cheng et al., 2016b). In our current study, the expression levels of CCL15 and CXCL7 (PPBP) were decreased in cancer tissues compared with the adjacent normal tissues. Bodelon et al. reported that the serum chemokine CCL15 correlated with poor prognosis in early-stage lung cancer (Bodelon et al., 2013). Qiang et al. proved that the plasma level of CXCL7 was increased in LUSC patients compared to controls (Du et al., 2018). Our results were inconsistent with those of the aforementioned previous studies, which indicated that serum chemokines and tissue chemokines may play different roles in tumors. We hypothesize that the microenvironment plays a critical role in influencing gene function. Consequently, the effect of a specific gene may differ between normal and tumor tissues, and this difference may be especially important for chemokines. Furthermore, alternative proteins encoded by the same gene may have widely divergent functions depending on the microenvironment. However, few studies have examined the potential association between CXCL7 and CCL15 and the corresponding prognosis of LUSC patients. The pathogenic mechanisms of these chemokines in LUSC remain unclear and require further investigation.

Increasing evidence suggests that the chemokine signaling pathway can reshape the tumor immune microenvironment *via* the chemotactic migration of infiltrating immune cells and regulation of angiogenesis (Sokol and Luster, 2015; Nagarsheth et al., 2017). In this study, we compared immune cell infiltration between the high-risk and low-risk groups and observed that the high-risk group displayed a higher immune infiltration density, including tumor-infiltrating immune cells and immune-related genes. Furthermore, the antigen-presenting ability and T-cell activation were elevated in the high-risk group. These results proved the immune activity characteristics of the chemokine signaling pathway in LUSC. However, the abundance of CD8 T-cells was increased in the low-risk group, whereas Treg cell abundance was decreased in this group. These findings could partly explain why patients in the low-risk group had a better prognosis. The PD-L1, TMB, and MSI statuses are widely used as immunotherapy biomarkers to identify patients who may respond to immunotherapy (Chan et al., 2019; Luchini et al., 2019). Our results showed that the expression levels of TMB and PD-L1 did not significantly differ between the two groups, whereas the expression of CTLA4 was increased in the high-risk group. Interestingly, consistent with the immune cell infiltration analysis, patients in the high-risk group had a higher IPS for CTLA-4 inhibitors, indicating increased sensitivity to CTLA-4 inhibitors. These results suggested that our CSRG signature could reflect the tumor immune infiltration status and could also predict the response of LUSC patients to anti-CTLA4 therapy.

Despite significant progress in immunotherapy, cisplatin-based chemotherapy remains the standard treatment option for LUSC patients (Chaft et al., 2021). In this study, we observed that low-risk patients presented higher sensitivity to cisplatin, etoposide, and vinorelbine, which are widely used as first-line treatments for LUSC patients. Tumor stemness is widely known to potentially induce resistance to chemotherapy, which could explain why the low-risk group was more sensitive to chemotherapy (Liu et al., 2021; Pan et al., 2021). In addition, we also found that patients in the low-risk group were more sensitive to first- and second-generation EGFR tyrosine kinase inhibitors. Moreover, the lack of an effective therapeutic target correlated with poor survival in LUSC. Clinicians could use the CSRG signature as a tool to predict sensitivity to chemotherapy and targeted therapy before treatment and avoid overtreatment or side effects in nonresponder patients.

However, this study was also subjected to several inevitable limitations that should be addressed. First, all the cohorts in our study were downloaded from public databases, and the results need to be verified by large-scale clinical studies. Second, the molecular mechanisms of these genes in this signature should be further validated in future experiments.

## Conclusion

Taken together, our study investigated the role of chemokine signaling-related genes in LUSC patients with stage I–III disease. We developed and validated a CSRG-related signature for the prognosis of LUSC patients. A prognostic nomogram combining clinical stage, T stage, and risk score was established for clinical OS prediction. More importantly, the signature could serve as a prediction tool for clinicians to predict sensitivity to immuno-chemotherapy and target therapy. Our study provides insight into the multifaceted role of the chemokine signaling pathway in LUSC and may help clinicians implement optimal individualized treatment for patients.

## Data Availability

The original contributions presented in the study are included in the article/Supplementary Material. Further inquiries can be directed to the corresponding authors.
