# Supplementary material for: Determination of a prediction model for therapeutic response and prognosis based on chemokine signaling-related genes in stage I–III lung squamous cell carcinoma
Source: Front Genet. 2022 Aug 31;13:921837. doi: 10.3389/fgene.2022.921837 (PMC9470854; doi:10.3389/fgene.2022.921837)
Supplement: Supplementary file 1 [file DataSheet1.docx]

Supplementary Material

## Supplementary Figures


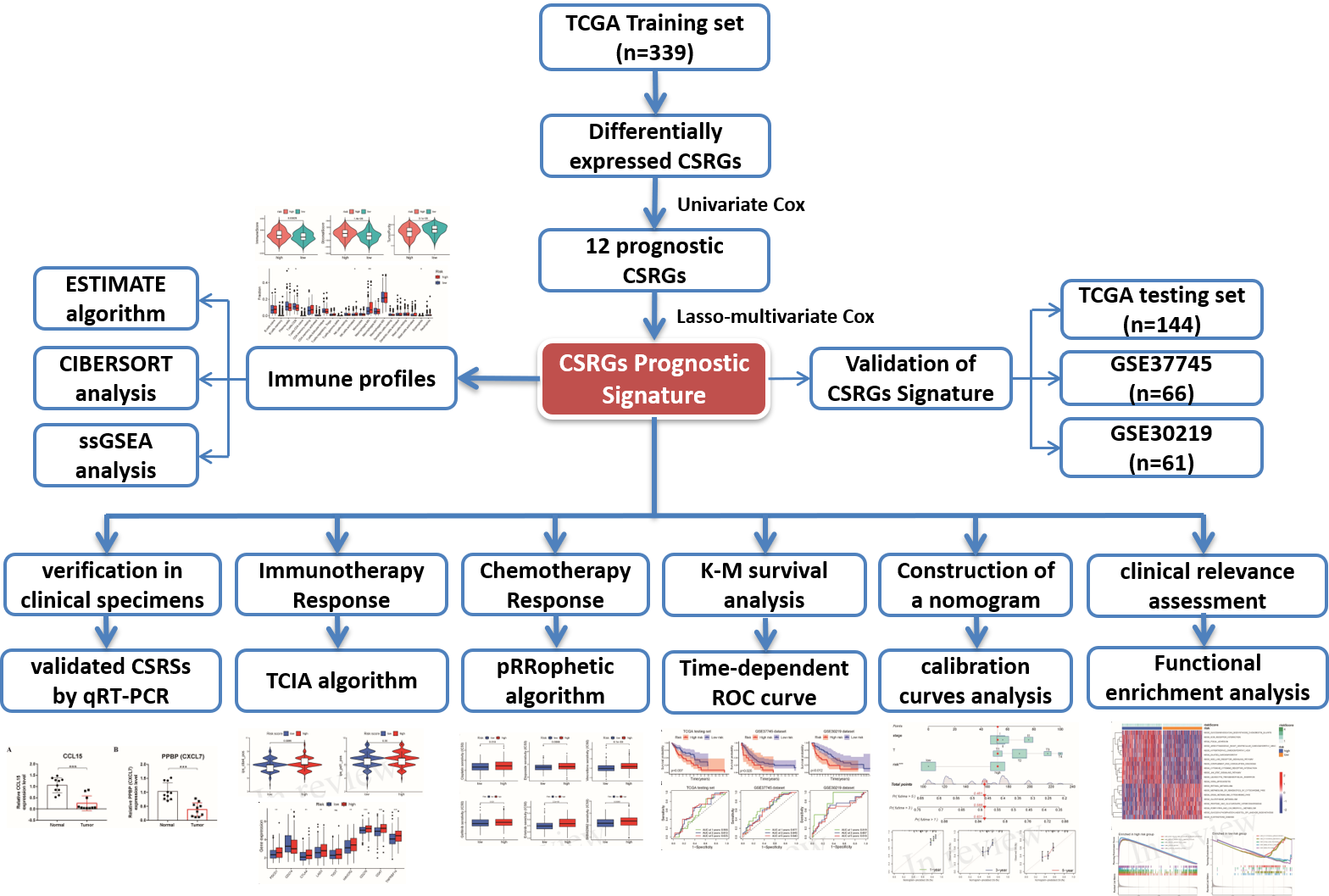


**Supplementary Figure 1 | The detailed construction process of this study.**

**
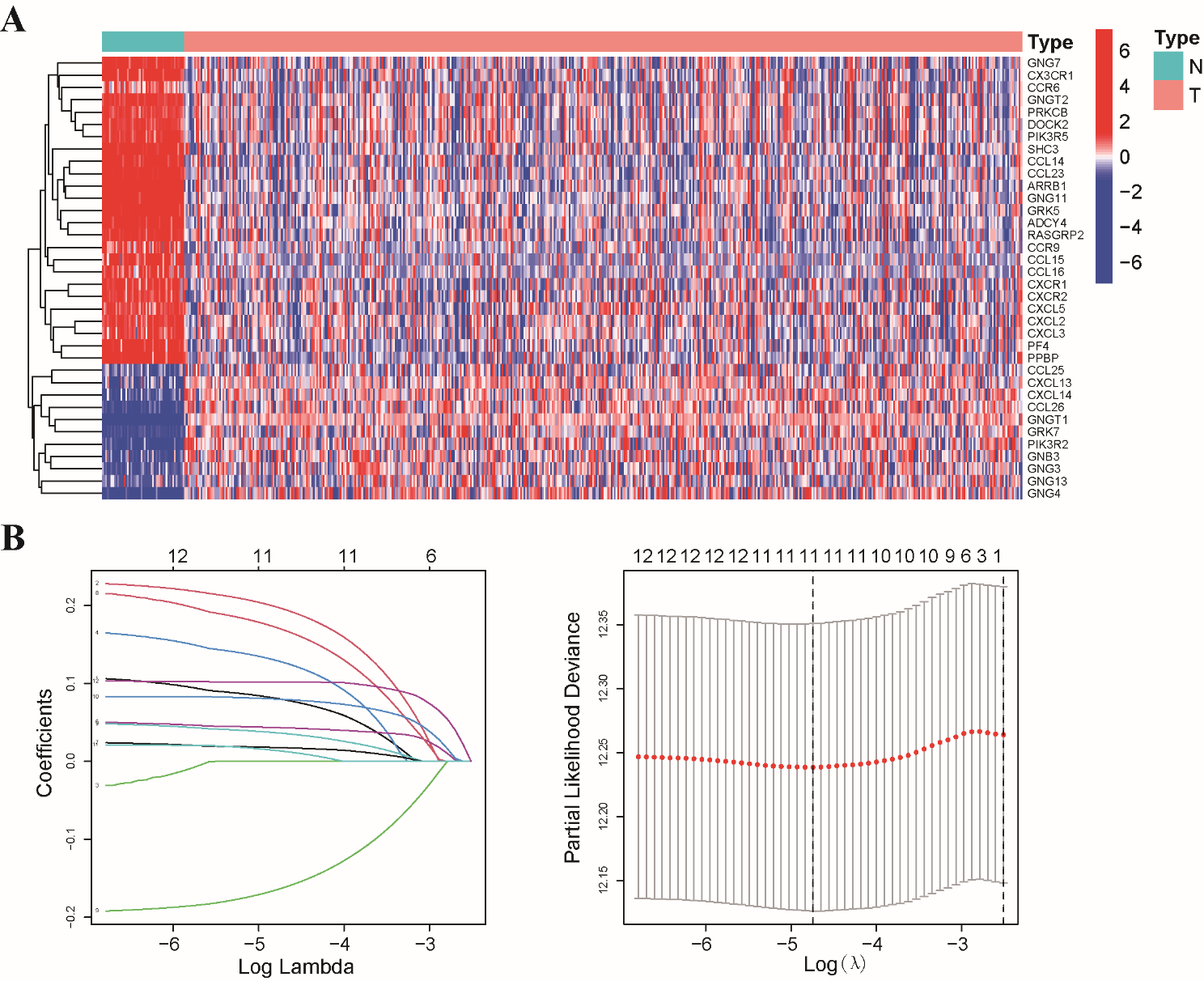
**

**Supplementary Figure 2 | (A)** The LASSO regression analysis was utilized to select most useful prognostic genes **(B)** Heatmap of differentially expressed CSRGs between tumor and normal tissues.


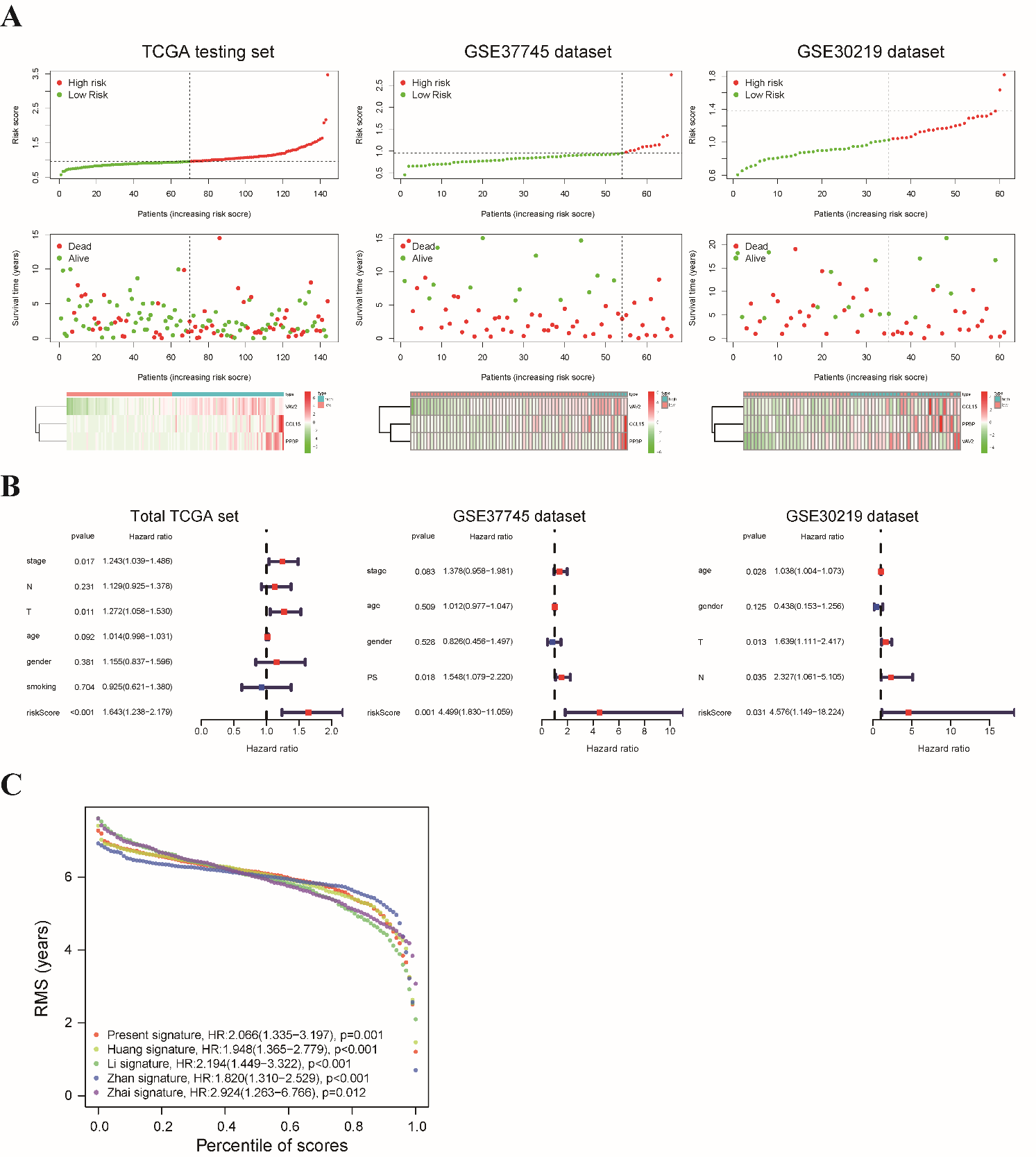


**Supplementary Figure 3 | (A)** The distribution of risk scores, survival status and genes expression panel in the validation set. **(B)** Univariate Cox regression analyses of the risk score in the total TCGA cohort and GSE37745, GSE30219 external validation cohort. **(C)** The RMS time curves for our signature and other 4 prognostic signatures.

**TABLE S1 |** The list of 185 chemokine signaling related genes.

| ADCY1 | CCL5 | CXCR5 | IKBKB | PRKCD |
| --- | --- | --- | --- | --- |
| ADCY2 | CCL7 | ACKR3 | IKBKG | PRKCZ |
| ADCY3 | CCL8 | CXCR6 | ITK | PRKX |
| ADCY4 | CCR1 | DOCK2 | JAK2 | PTK2 |
| ADCY5 | CCR10 | ELMO1 | JAK3 | PTK2B |
| ADCY6 | CCR2 | FGR | KRAS | PXN |
| ADCY7 | CCR3 | FOXO3 | LYN | RAC1 |
| ADCY8 | CCR4 | GNAI1 | MAP2K1 | RAC2 |
| ADCY9 | CCR5 | GNAI2 | MAPK1 | RAF1 |
| AKT1 | CCR6 | GNAI3 | MAPK3 | RAP1A |
| AKT2 | CCR7 | GNB1 | NCF1 | RAP1B |
| AKT3 | CCR8 | GNB2 | NFKB1 | RASGRP2 |
| ARRB1 | CCR9 | GNB3 | NFKBIA | RELA |
| ARRB2 | CDC42 | GNB4 | NFKBIB | RHOA |
| BCAR1 | CHUK | GNB5 | NRAS | ROCK1 |
| BRAF | CRK | GNG10 | PAK1 | ROCK2 |
| CCL1 | CRKL | GNG11 | PARD3 | SHC1 |
| CCL11 | CSK | GNG12 | PF4 | SHC2 |
| CCL13 | CX3CL1 | GNG13 | PF4V1 | SHC3 |
| CCL14 | CX3CR1 | GNG2 | PIK3CA | SHC4 |
| CCL15 | CXCL1 | GNG3 | PIK3CB | SOS1 |
| CCL16 | CXCL10 | GNG4 | PIK3CD | SOS2 |
| CCL17 | CXCL11 | GNG5 | PIK3CG | STAT1 |
| CCL18 | CXCL12 | GNG7 | PIK3R1 | STAT2 |
| CCL19 | CXCL13 | GNG8 | PIK3R2 | STAT3 |
| CCL2 | CXCL14 | GNGT1 | PIK3R3 | STAT5B |
| CCL20 | CXCL16 | GNGT2 | PIK3R5 | TIAM1 |
| CCL21 | CXCL2 | GRB2 | PLCB1 | TIAM2 |
| CCL22 | CXCL3 | GRK1 | PLCB2 | VAV1 |
| CCL23 | CXCL5 | GRK4 | PLCB3 | VAV2 |
| CCL24 | CXCL6 | GRK5 | PLCB4 | VAV3 |
| CCL25 | CXCL8 | GRK6 | PPBP | WAS |
| CCL26 | CXCL9 | GRK7 | PREX1 | WASL |
| CCL27 | CXCR1 | GSK3A | PRKACA | XCL1 |
| CCL28 | CXCR2 | GSK3B | PRKACB | XCL2 |
| CCL3 | CXCR3 | HCK | PRKACG | XCR1 |
| CCL4 | CXCR4 | HRAS | PRKCB | CXCL17 |

**TABLE S2 |** Sequences of the primer used for qRT-PCR

| mRNA | Forward primer (5'-3') | Reverse primer (3'-5') |
| --- | --- | --- |
| CCL15 | CTGACTGCTGCACCTCCTACATC | GACCACTGGGTTTGGCACAGAC |
| PPBP(CXCL7) | GCAACCAAGTCGAAGTGATAGCCA | ATCAGCAGATTCATCACCTGCCAA |
| GAPDH | CCAACTGCCAGACTACCAC | GGACCAGGCTGTTCCAAGA |

**TABLE S3 |** List of tools used in this study

| Tools | Input | Output | R package | Description |
| --- | --- | --- | --- | --- |
| Nomogram | Risk score and clinicopathological characteristics | nomo risk value and Nomogram | "survival", "regplot" and | a diagram representing the relations between three or more variable quantities by means of a number of scales |
| ESTIMATE | RNA expression data | tumor purity, stromal score, immune score and ESTIMATE score | ESTIMATE R package | a tool for predicting tumor purity, and the presence of infiltrating stromal/immune cells in tumor tissues |
| ssGSEA | RNA expression data and 29 immunity-related signatures | 29 immunity-related functions, pathways and immune cell types | "GSEAbase” and “GSVA” R package | to comprehensively assess the immunologic characteristics of every sample |
| CIBERSORT | RNA expression data | fractions of immune cell population | CIBERSORT R package | an analytical tool from the Alizadeh Lab developed by Newman et al. to provide an estimation of the abundances of member cell types in a mixed cell population |
| pRRophetic | RNA expression data | drug sensitivity (IC50) values | pRRophetic R package | an R package for prediction of clinical chemotherapeutic response from tumor gene expression levels |
